# Critical care analytes: pre-analytical factors affecting result quality for combined blood gas and electrolyte systems

**DOI:** 10.1155/S1463924689000404

**Published:** 1989

**Authors:** R. F. Moran, A. Feuillu

**Affiliations:** 1 Technical Liaison Ciba-Corning Diagnostics Medfield Massachusetts 02052 USA; 2 Centre Hospitalier Régional Pontchaillon Laboratoire de Services d'Urgences et de Réanimation Rennes 35033 France


Journal of Automatic Chemistry Vol. 11, No. 5 (September–October 1989), pp. 201–205

### Critical care analytes: pre-analytical factors affecting result quality for combined blood gas and electrolyte systems

**R. F. Moran**
*Technical Liaison, Ciba-Corning Diagnostics, Medfield, Massachusetts 02052, USA*

**and A. Feuillu**
*Centre Hospitalier Régional Pontchaillon, Laboratoire de Services d'Urgences et de Réanimation, Rennes 35033, France*

### Introduction

'Incorrect test results are much more often due to patient and specimen collection considerations than to laboratory variability or error' [1], according to one USA based professional organization. Shapiro [2] noted in a study involving thousands of patients, more than 5% of the individually collected specimens were questionable based on pre-analytical error, and, indeed, more than 15% of the specimens received from the emergency room were associated with significant pre-analytical error! Since 'blood gas and pH analysis has more immediacy and potential impact on patient care than any other laboratory determination' [3], it is evident that while the medical laboratory analyst must be primarily aware of the impact of analytical error, pre-analytical considerations need to be taken into account by others in the assessment of the reliability of results to be reported. An understanding by physicians, nurses and other direct patient care practitioners of the 'Cycle of quality assurance' (see figure) for blood gases and other critical analytes will minimize the chances that pre-analytical error will significantly effect patient care. The laboratory professional must be involved in the process of education about these issues.

### Pre-analytical considerations

Of major impact on the utility of blood gas, total haemoglobin and electrolyte results are (1) the physiologic stability of the patient; (2) the effects of blood metabolism and collection/transport conditions on the analytes measured; and (3) the timeliness of the collection and reporting of results.

A comprehensive review of various pre-analytical aspects of blood gas analysis is given in a document prepared by the USA-based National Committee for Clinical Laboratory Standards (NCCLS) [3]. This article concentrates on certain key points of that document, as well as providing some more recent additional information.

### Physiologic stability

The ventilatory process is a key consideration affecting blood gas values. Patient postural changes affect ventilatory rate and depth, thus causing real changes in blood gas values, especially in the $p_{CO_2}$(aB), but also in the $p_{O_2}$(aB). Circulatory compromise, such as that seen in arrested patients during resuscitative efforts, can cause samples from peripheral collection sites to be non-representative of whole body conditions. The effects of these types of conditions could either be recognized by the clinician or the changed results assumed to be 'laboratory' error, thus masking a need for intervention, or bringing about inappropriate intervention. Patients on controlled ventilation, or supplemental oxygen, ideally should be assessed only after new settings have been in effect for at least 20 min. In other cases, especially when 'weaning' a patient from mechanical ventilation (PEEP, CPAP, IMV) or supplemental oxygen, a result may be urgently required between 5 and 10 min after each change.

### Collection and transport conditions

The collection process itself, being potentially painful, may bring on an apprehensive hyperventilation by the patient, causing an immediate effect on $p_{CO_2}$(aB), lowering the value.

Collection devices are also subject to limitation. The ideal system incorporates a collection device (syringe) that minimizes or eliminates exposure to air, includes the correct extent of heparin anticoagulation (20–50 U/ml of blood) and enables identification of the collection site as being arterial (by responsiveness to arterial pressure). If subsequent or simultaneous measurement of sodium, potassium or ionized calcium on the same sample is anticipated, the effects of the anticoagulant must be taken into account. (For example the use of sodium heparinate will normally elevate the measured sodium by 1–3 mmol/l, or even more if the syringe is not completely filled. Also, the binding effects of heparin on ionized calcium can lower the sample's ionized calcium significantly if the heparin dose is >20 U/ml of blood.)

One of the more significant sources of unrecognized error in the $p_{CO_2}$ measurement is the use of too great a *volume* (as opposed to *units*) of liquid heparin relative to the amount of blood collected (underfilling a syringe), which leads to a falsely low value. The pH may also be lowered,

0142–0453/89 $3.00 © 1989 Taylor & Francis Ltd.

but only when the underfilling is severe. Use of a low-dose dried lithium heparin anticoagulant or a 'calcium balanced', or 'titrated', heparin should eliminate these problems.

Exposure of the sample to air has the obvious effects on both $p_{O_2}$ and $p_{CO_2}$ due to potential differences in the tensions of the gases in air versus the sample. An unrecognized cause of error is the common practice of 'flicking' the syringe to rid it of air bubbles prior to measurement, since this excessive agitation can cause formation of tiny air bubbles which will equilibrate with the sample more readily than a large bubble. This will typically lower $p_{CO_2}$ and elevate $p_{O_2}$ values; however, if the patient is breathing high concentrations of oxygen, air contamination may cause a lowering of $p_{O_2}$ values.

The use of 'arterialized' capillary blood for the measurement of $p_{O_2}$ should be recognized as a last resort only. While acceptable for pH and $p_{CO_2}$, the results are a reliable estimate of arterial values only at oxygen tensions less than 8·0 kPa (60 mmHg). Above that level, the opposing effects of tissue metabolism and air contamination during collection create a large potential uncertainty in results at oxygen tensions where accuracy and precision are paramount. (For example at or near 13·0 kPa [approximately 100 mmHg], the point at which oxygen therapy levels are most critical in neonates.)

Vacuum tubes are suitable collection devices only for venous blood and acid base/electrolyte measurement, since, because of the vacuum, they aspirate blood from any vessel, and thus reduces the ability to assess placement of the needle in an artery. The gas bubble in the tube can change sample results (lower $p_{CO_2}$ and elevate $p_{O_2}$). If a vacuum tube is used the report should be labelled as to source, and the appropriate symbol used [for example pH(vB), $p_{CO_2}$(vB)].

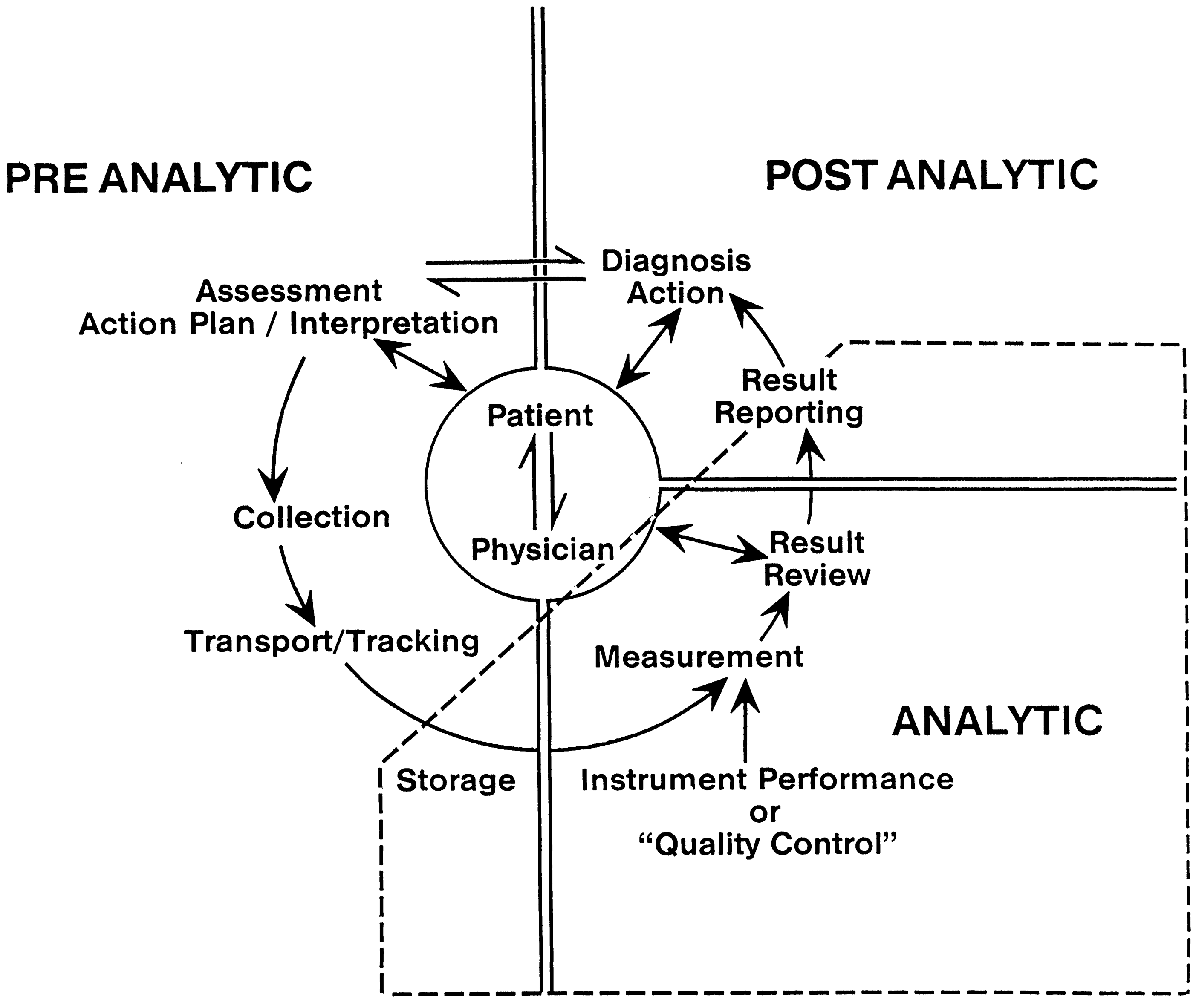

*The cycle of quality assurance. (The area enclosed by the dashed line represents that for which the laboratory typically assumes responsibility.)*

© 1990 R. F. Moran

Due to frequency of multiple blood gas analyses on the same patient over short time intervals, recording of the exact collection time on each specimen is necessary. If more than one specimen on the same patient is in the blood gas laboratory simultaneously without the collection time or sequence being labelled, the results should be held from reporting until the issue is resolved.

The immediate impact that blood gas results can have on clinical management and decisions affecting morbidity and mortality emphasizes the need for strict adherence to proper identification of the patient, patient conditions and the sample while at the same time treating the sample as quickly as possible.

### Metabolic effects

Once collected, the specimen should be analysed in a few minutes (less than 10) or placed in an ice-water slurry. (Use of crushed ice alone will cause uneven cooling and artificial coolants may cause freezing of portions of the blood sample.) The effect of this slurry is to reduce cellular metabolism which in turn minimizes the utilization of oxygen and production of carbon dioxide by this 'living tissue' sample. A combination of *immediate* icing and analysis as soon as possible may be required with known severe leucocytosis (leukaemia) or reticulocytosis, or with severe sepsis, since the increased numbers of metabolically active cells can cause rapid changes in the gases, especially oxygen.

If simultaneous or subsequent analysis of potassium on the same sample is anticipated, the analysis should occur within 30 min of collection and placement in the ice-water slurry. Potassium values on samples kept in an ice-water slurry for longer times may be elevated by 0·2 mmol/l or greater [6]. Some data indicates that samples from patients with cold agglutinins may be substantially elevated after only a few (<15) minutes in the ice-water slurry [7]. Both of these findings were observed when no visible haemolysis was present.

### Turnaround time

While post-collection intervals as long as two hours may not invalidate the measurement of pH, $p_{O_2}$ and $p_{CO_2}$ in a properly iced sample, most clinical needs are for a much shorter duration (15 to 30 min turnaround time) [8].

### Analyser maintenance

For consistently reliable results, it is important that all routine maintenance be carried out in the manner and frequency specified by the manufacturer. While changes in maintenance may not affect results immediately, substantial changes in practice will, over time, alter performance. Changes in performance may also result if reagents and solutions other than those specified by the manufacturer are used. Again, the changes may be subtle and may take a long time to become obvious. The end result, however, will be the failure of the analyser to perform as reliably as it is capable of doing.

### Analysis

When introducing the sample to the analyser, it is essential to carefully read and follow the instructions for the particular instrument. Each analyser's sample-handling protocol may be different, and differences in technique on the same analyser may affect results. Complete mixing of the sample prior to analysis, by a combination of rolling and inversion of the sample container, is important to ensure satisfactory results in the measurement of pH, $p_{CO_2}$ and $p_{O_2}$, and it is critical if the measurement of total haemoglobin (tHb) or packed cell volume (pcv or Hct) is to be performed.

### Reporting results

After analysis, results for each analyte should be compared with the history, patient temperature, past results, ventilatory/supplemental oxygen conditions at the time of collection, and with prior 'blood gas' values on the same patient to see if they are compatible. An excellent check for within-sample result consistency is application of the modified 'alveolar air equation':

$$p_{O_2}(A)^* = F_IO_2[p_{atm} - p_{H_2O}] - 1{\cdot}25[p_{CO_2}(aB)] \quad (1)$$

* (A) refers to alveolar gas.

By substitution of the fraction of inspired oxygen ($F_IO_2$), the atmospheric pressure ($p_{atm}$), the water vapour pressure $p_{H_2O}$) and the measured $p_{CO_2}$(aB), the alveolar partial pressure of oxygen can be estimated and compared with the arterial value as measured. Given the expected difference between alveolar and arterial $p_{O_2}$, [A–$aDO_2$], an assessment of 'mistakes' in the measurement can be made before reporting the results. The minimum criterion is that the A-$aDO_2$ be >0, although typically it is 0·7–2 kPa.

The usual precautions hold true in reporting blood gas results by whatever means (verbal, written or automatic) but again, as with the patient and specimen identification, the immediate and critical impact of the blood gas results serves to emphasize that *no* exceptions to standard protocol can be tolerated.

### Temperature 'correction' of blood gas values

The values for pH and gas tensions unquestionably vary with respect to temperature. As a result, many laboratories report blood gas values 'corrected or adjusted' to the temperature of the patient.

While some clinicians may have practical experience and 'know' what to expect at temperatures other than 37 °C, there are no widely accepted reference values at different temperatures. Next, is the issue of what to do with the 'adjusted' values obtained. For example, does one try to regulate the patient's pH and $p_{CO_2}$ to 7·40 and 5·33 kPa (40 mmHg) respectively? Finally, what algorithms does one choose to use to make the adjustment from 37 °C to the patient's temperature?

From the perspective of the blood gas laboratory, the first two issues are most reasonably addressed by accepting the recommendations of Ashwood *et al.* [9]. For the acid-base values (pH and $p_{CO_2}$) and calculated quantities, such as actual bicarbonate, report values at the measuring temperature of 37 °C. For $p_{CO_2}$ and $p_{O_2}$, used in assessing gas exchange and possibly compared with expired gases, report temperature 'adjusted' values. On a practical basis, this means that the carbon dioxide tension and oxygen tension values should be reported at both temperatures, while pH should be reported only for 37 °C. The report itself should clearly distinguish between the values at the two temperatures.

The last issue is easiest to address, since, for most blood gas laboratories, the algorithm used is that chosen by the manufacturer of the analyser. NCCLS document C-12T2 [4], a frame of reference accepted by many, states that its standard is met if the algorithm used to make the 'correction' gives results that are quantitatively similar to those specifically documented in the standard. Algorithms chosen by the three major blood gas manufacturers meet those criteria.

### Assessment of measuring system performance: quality control

A properly maintained blood gas analyser, calibrated either with buffers and gases prepared by the manufacturer or traceable to them [10], will give years of reliable service.

As noted earlier, many or even most blood gas result 'errors' occur outside the area of laboratory analysis [1, 2]. Additionally, many modern blood gas analysers incorporate automatic devices which signal when the analyser is not performing adequately.

Despite these facts, there is an increasing level of activity in the use of internal and external quality control schemes to check on analyser performance. Quality control schemes, of course, only check on performance of the analyser on the test material actually chosen, and at the time the control material is actually measured. As such, any such quality control scheme needs to be considered as only a part of a complete programme that addresses the various issues discussed in this report. The requirements and complications of quality control protocols for a blood gas analyser are substantially different from other analyses performed in the clinical laboratory environment. This is due substantially to the combination of turnaround time requirements for the patient sample, the fact that the patient sample is fresh whole blood—that is—living tissue, and of course, the various pre-analytical items discussed earlier in this report.

The optimum technique for establishing the inaccuracy and imprecision of the gas channels of an individual blood gas analyser is the use of whole blood tonometry [11, 12], using fresh anticoagulated whole blood, carefully equilibrated with a known standard mixture of oxygen, carbon dioxide and nitrogen at 37 °C. Properly done, this technique enables determination of the performance of the analyser when measuring blood at the partial pressures of the tonometry gas mixtures. In essence, tonometry more closely resembles a primary standard in a true sample matrix, not merely a control material. The technical and economic advantages of whole blood tonometry must be balanced by hazard potential and the labour-intensive nature of the process.

The alternative to whole blood tonometry is the use of commercially available pre-packaged materials, each type having its own 'mix' of advantages. There are currently three major types of commercially available controls used for blood gas quality control each of which is gas-equilibrated; aqueous buffer solutions, blood-based (haemoglobin-containing) materials, and perfluorocarbon/oil emulsions. Their effectiveness varies and is dependent on the particular analyte and the physical and chemical characteristics of the material itself. While, for blood gases alone, one might consider the purported marginal advantages of non-aqueous materials, the fact is that aqueous buffers are economical and acceptable for routine performance assessment of pH and blood gas. However, if electrolytes are also to be measured on the same analyser as the blood gas, it is probably most feasible to use the aqueous-based materials, since acceptable values for the electrolytes are also available. While not as good as whole blood (patient) duplicates run on separate analysers offer the best combination of economy, feasibility and analytical quality.

The fundamental issue with respect to any non-blood control is that their physical and chemical properties do not, in many respects, match those of whole blood. As a result, they may not detect certain problems or they may signal problems that are not there. The former situation can result in clinical problems; the latter creates financial issues due to the cost of repeat controls, labour, and possibly, unnecessary service calls.

Selection of a particular type of control must be based on evaluation of its technical and other merits in the context of a complete blood gas quality control programme. In that context, the pre-packaged controls can serve a primary role in assessing blood gas analyser performance, while remembering that when a true performance issue arises (with respect to the reliability of patient results), only reference methods/materials may be able to settle it satisfactorily [12].

Duplicate analysis of the same blood sample can be a useful tool, but should not be used as the sole method for assessing instrument performance. Duplicates measured on two different analysers are most useful in detecting individual sample errors due to the improbability of similar errors occurring simultaneously on two different instruments. Certain types of pH system malfunction (reference circuit) can only be reliably detected using whole blood. In an emergency situation, where analytical reliability and turnaround time are equally important, duplicate analysis of this type may be a requirement. Bear in mind, however, that duplicate analysis on the same instrument has a marginal usefulness at best, and at worst, provides a false sense of security.

Descriptions of available blood gas control materials [12, 13] and detailed protocols for their use [3, 13] may assist in choosing the quality control programme that meets the requirement of the particular institution.

For those just beginning to consider a formal quality control programme for blood gas or any other analyte, it is best to assess the clinical requirements for result quality, the analyser's age and performance history, as well as the control scheme and materials available. While there is no substitute for quality, the extent needed for a particular analyte group, such as blood gas, is not the same in all institutions.

It is most important to start by reviewing all of the pre-analytical issues discussed above with the medical staff of the institution, at the same time determining their clinical requirements. Once a quality assurance programme to address the pre-analytical concerns has been established, then a quality control program for the analyser, sufficient to meet the clinical requirements, can be started.

Typically, this might begin by combining the use of (1) daily duplicate analysis of several blood specimens (preferably on two analysers), plus statistical comparison of results; and (2) weekly or daily analysis of buffers of a different lot number than that used to calibrate the analyser. In either case, if results of analysis differ to an unacceptable extent, corrective action must be taken and the results recorded. The next step, after some experience is gained with the above approach, might be to use a single level (abnormal—either high or low) of commercial pre-packaged control. This is used to test performance after each change in reagents or electrodes. An even better approach would be to use the control daily so that normal performance can be compared with performance after electrode or reagent changes.

In all cases, in developing a quality control programme, keep records so that a real value to the quality control scheme is clear. By observing values obtained, calculating mean values and standard deviation [13] and comparing results with specific performance problems, an overall scheme can be developed. If done in this planned, stepwise manner, it will best meet the particular institution's requirements by balancing costs, time, and quality.

### Summary

As indicated at the outset of this article, the critical nature of the clinical requirements when blood gas testing is indicated, coupled with the labile nature of the sample and rapidly changing patient conditions, makes it imperative that the analyst be aware of more than just the analysis itself. Knowledge by the analyst of the effects of the changing patient environment, and the manner in which the sample is treated in the few short minutes from collection to reporting are equally important if one is to assure timely and clinically meaningful results.

### References and Bibliography

1. Jones, R. J. and Paulonis, R. M. (Eds), *Laboratory Tests in Medical Practice* (AMA Council on Scientific Affairs, 1980), 11.
2. Shapiro, B. A., Harrison, R. A., Cane, R. D. and Templin, R., *Clinical Application of Blood Gases*, 4th edn. (Year Book Medical Publishers, Chicago, 1988).
3. Eichhorn, J. H., Moran, R. F. and Cormier, A. D., *Blood Gas Pre-Analytical Considerations: Specimen Collection, Calibration and Controls* (NCCLS Publication, C-27T, Villanova, 1989).
4. Burnett, R. W. *et al., Definitions of Quantities and Conventions Related to Blood pH and Gas Values* (NCCLS publication C-12T, Villanova, 1982).
5. Siggaard Anderson, O., *The Acid Base Status of the Blood* (Munksgaard, Copenhagen, 1978).
6. Moran, R. F. and Grenier, R. E., The effects of 'standard' blood gas transport and storage conditions on electrolyte and haemoglobin results. Presented at European Working group on Ion Selective Electrodes, 9th Meeting, Stresa, Italy (1988).
7. Misiano, D. (Massachusetts [USA] General Hospital), Personal communication (November 1988).
8. Vanderlinde, R. E., Goodwin, J., Koch, D., Scheer, D., Steindel, S. and Cembrowski, G., *Guidelines for Providing Quality Stat Laboratory Services* (AACC Press, 1987).
9. Ashwood, E. R., Kost, G. and Kenny, M., *Clinical Chemistry* **29**, (1985), 1877.
10. Terra, P., Maley, T. C. and Moran, R. F., *ASEAN Journal of Clinical Science*, Supplement (1984), 23–29.
11. Fallon, K., Burnett, R. W., Christiansen, T. F., Clausen, J. L., Durst, R. A., Ehrmeyer, S., Eichhorn, J. H., Ladenson, J. L., Legg, K. D., Moran, R. F., VanKessel, A. L. and Weisberg, H. F. *Devices Measuring $PO_2$ and $PCO_2$ in Blood Samples* (NCCLS Press, C-21T, p. 130.
12. Hansen, J. E., Stone, M. E. Ony, S. T. and VanKessel, A. L., *American Review of Respiratory Disorders*, **125** (1982), 480.
13. Moran, R. F. and Grenier, R. E., *Canadian Journal of Medical Technology*, **50** (1988), 95.

